# A Biofouling Resistant Zwitterionic Polysulfone Membrane Prepared by a Dual-Bath Procedure

**DOI:** 10.3390/membranes12010069

**Published:** 2022-01-04

**Authors:** Irish Valerie B. Maggay, Hana Nur Aini, Mary Madelaine G. Lagman, Shuo-Hsi Tang, Ruth R. Aquino, Yung Chang, Antoine Venault

**Affiliations:** 1 R&D Center for Membrane Technology and Department of Chemical Engineering, Chung Yuan Christian University, Chung-Li 32023, Taiwan; irish.maggay@gmail.com (I.V.B.M.); hananuraini96@gmail.com (H.N.A.); steventang0321@cycu.org.tw (S.-H.T.); 2 School of Chemical, Biological, and Materials Engineering and Science, Mapúa University, 658 Muralla St., Intramuros, Manila 1002, Philippines; marymlagman@gmail.com (M.M.G.L.); rraquino@mapua.edu.ph (R.R.A.)

**Keywords:** polysulfone membrane, zwitterionic modification, antifouling membrane, blending

## Abstract

This study introduces a zwitterionic material to modify polysulfone (PSf) membranes formed by a dual bath procedure, in view of reducing their fouling propensity. The zwitterionic copolymer, derived from a random polymer of styrene and 4-vinylpyrridine and referred to as zP(S-*r*-4VP), was incorporated to the PSf solution without any supplementary pore-forming additive to study the effect of the sole copolymer on membrane-structuring, chemical, and arising properties. XPS and mapping FT-IR provided evidence of the modification. Macrovoids appeared and then disappeared as the copolymer content increased in the range 1–4 wt%. The copolymer has hydrophilic units and its addition increases the casting solution viscosity. Both effects play an opposite role on transfers, and so on the growth of macrovoids. Biofouling tests demonstrated the efficiency of the copolymer to mitigate biofouling with a reduction in bacterial and blood cell attachment by more than 85%. Filtration tests revealed that the permeability increased by a twofold factor, the flux recovery ratio was augmented from 40% to 63% after water/BSA cycles, and irreversible fouling was reduced by 1/3. Although improvements are needed, these zwitterionic PSf membranes could be used in biomedical applications where resistance to biofouling by cells is a requirement.

## 1. Introduction

Polysulfone (PSf) is one of the most commonly used materials in membrane technology [1]. Its chemical, thermal, and mechanical resistance, combined with its excellent hydrolytic stability and relatively inexpensive production costs, make it ideal for widespread use in membrane fabrication. It has been employed as the main membrane matrix material in a wide range of applications including: ultrafiltration for water treatment [2], gas separation [3], desalination via membrane distillation [4], hemodialysis [5], etc.

PSf membranes are commonly prepared by phase-inversion processes including wet-immersion [6,7,8], vapor-induced phase separation [4,9], or dry–wet phase inversion [10,11] because they are easily carried out, require relatively inexpensive equipment, and are reproducible and so are scalable.

However, the intrinsic hydrophobicity of polysulfone makes PSf membranes prone to fouling. In particular, biofouling can readily occur when the membranes come into contact with an aqueous environment. Thus, polysulfone membranes used in wastewater treatment, blood filtration, or any environment containing proteins, micro-organisms, or cells have to undergo a modification procedure in order to improve their water permeability and/or reduce their interactions with biofoulants [12].

Over 20 years ago, Whitesides’ group presented some guidelines to prepare surfaces to resist the adsorption of proteins from liquids [13]. These procedures can be extended to the fabrication of anti-biofouling membranes. The formation of a protective hydration layer at the interface between the membrane and its surrounding environment is the key. This can be realized using a large variety of hydrophilic or amphiphilic materials incorporated into the membrane system via different surface or bulk modification techniques [14]. Some effective anti-biofouling materials are the PEGylated materials, derived from poly(ethylene glycol) (PEG) and the zwitterionic materials [15]. The latter are regarded as even more effective to efficiently trap water [16]. These materials can be included in membrane systems by coating (or physical adsorption) [17], grafting (or chemical adsorption) [18], or *in-situ* modification (also called the blending method) [19]. As mentioned above, PSf is a common material in membrane technology, thus the body of literature on the development of antifouling PSf membranes is quite important. Recently, Hou et al. blended a poly(ethyleneoxide)-grafted amphiphilic copolymer with PSf to develop renewable antibacterial and antifouling membranes [20]. In a study published this year, Zhong et al. investigated the preparation of hemodialysis membranes made of PEGylated PSf, and demonstrated the effect of PEG chains on the hydrophilicity and hemocompatibility of the membranes [21]. Yu et al. grafted a copolymer referred to as [3-(methacryloylamino)propyl]-dimethyl (3-sulfopropyl) ammonium hydroxide on the surface of PSf membranes to improve their resistance to bovine serum albumin fouling [22]. Yue et al. used poly(sulfobetaine methacrylate) (PSBMA) to modify PSf membranes via surface-initiated atom transfer radical polymerization in order to improve their blood compatibility, cytocompatibility, and biofouling resistance [23]. The same zwitterionic material, PSBMA, was used by Xiang et al. for the surface modification of PSf membranes to improve their antifouling properties [24]. They concluded that PSBMA associated with poly(sodium methacrylate) “showed a synergistic effect in the process of coagulation” . In order to further maximize the grafting of SBMA on PSF membranes, Shahkaramipour et al. recently proposed to co-deposit the zwitterionic material with dopamine on the surface of PSf membranes [25].

Polymeric materials are not the only option to improve the antifouling properties of PSf membranes, and nanoparticles have also been proven to be a viable alternative to polymers [26,27,28,29], but the stability of these composite membranes can be questioned. Additionally, the vast majority of studies on the preparation of zwitterionic PSf membranes mention a surface modification process (grafting, coating). Very few studies, to our knowledge, have focused on the development of zwitterionic antifouling polysulfone membranes by an *in-situ* modification method. Yet this one-step membrane preparation and modification method is highly effective to readily prepare anti-biofouling porous membranes, as proven with poly(vinylidene fluoride)-based membranes [30]. The challenge of preparing zwitterionic copolymers compatible with hydrophobic membrane material needs to be tackled, in order to take advantage of both the exceptional properties of these materials and of the *in-situ* modification methods that allow fast membrane preparation, good stability, and easy scale-up.

**Figure 1:**
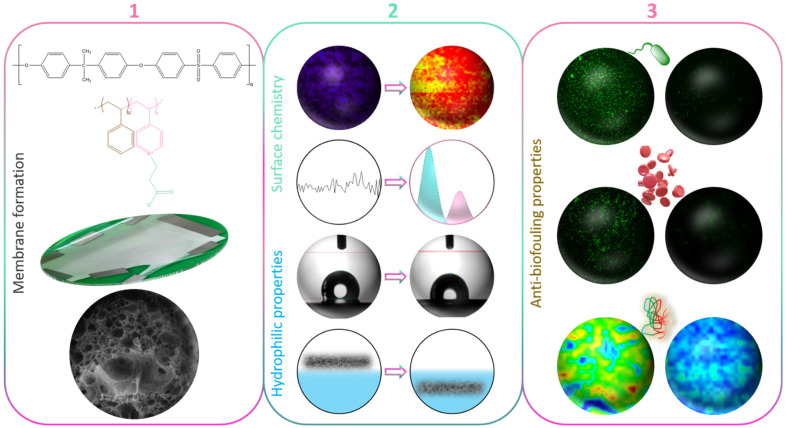
Schematic of the objectives of the work (**1**) to form zwitterionic polysulfone membranes by *in-situ* modification and rationalize membrane structure formation; (**2**) to characterize the changes in surface chemistry and surface/bulk hydration; (**3**) to evaluate the effect of the zwitterionic copolymer on biofouling using bacteria, blood cells, or proteins.

In this study, we used a copolymer made of styrene and a 4-vinylpyridine unit that then underwent a zwitterionization reaction using iodomethane. Although never used so far in the design of PSf membranes, the zwitterionic copolymer can be solubilized with polysulfone. Thus, it is a potential effective material for reducing the fouling propensity of PSf membranes prepared by *in-situ* modification. The experiments carried out in the frame of this work aimed primarily at testing this hypothesis by preparing novel zwitterionic membranes by liquid-induced phase separation. Although we have extensively evaluated the biofouling properties of the zwitterion copolymer in our previous works, modifying PSf membranes by blending zP(S-*r*-4VP) indicates the versatility of the copolymer in terms of being able to be solubilized in various hydrophobic polymeric matrices. As a first objective (Figure 1), the effect of the sole copolymer on membrane structures was tentatively rationalized. Thus, no other additive was incorporated into the membrane system, unlike in many studies reporting the use of pore-forming agents to fabricate UF/MF PSf membranes [31,32,33]. Subsequently, the focus was shifted on the complete characterization of the membranes’ physicochemical and wetting properties. At last, fouling was assessed using a large variety of biofoulants including proteins, bacteria, and whole blood. We hope to demonstrate that the zwitterionic derivative of poly(styrene-co-4-vinylpyridine) has great potential as an antifouling material for polysulfone membranes prepared by phase inversion.

## 2. Materials and Methods

### 2.1. Materials

Styrene monomers and 4-vinlypyridine monomers with a purity greater than 99% and 95%, respectively, were both purchased from Sigma-Aldrich, St. Louis, MO, USA. 2,2′-Azobis(isoburyonitrile) (AIBN, ≥99.7%) was bought from UniRegion Bio-Tech, Hsinchu, Taiwan. N,N-dimethylformamide (≥99.9%) (DMF) and N,N-dimethylacetamide (≥99.9%) (DMAC) were used as solvent for the copolymer synthesis and were purchased from Duksan Pure Chemicals Co., LTD, Ansan, South Korea. Acetone (≥99.9%) was also bought from Duksan Pure Chemicals Co., LTD, and used as a polarity modifier. Zwitterionization of the copolymer was performed using 3-iodopropionic acid (IPA, 99%) obtained from Alfa Aesar, Massachusetts, USA. Methanol and toluene, both used as nonsolvents during the copolymer synthesis were purchased from Avantor Macron Fine Chemicals, Pennsylvania, USA. Amberlite^®^ IRA-410 chloride form resin was obtained from Sigma-Aldrich and used to purify the copolymer by removing iodine.

The copolymer analysis involved the use of d-chloroform (99.8 atm%D) and tetrahydrofuran (inhibitor free) (THF) purchased from Sigma Aldrich and Tedia, Ohio, USA. respectively. Polysulfone, with an average molecular weight Mw of 50 kDa, was purchased from Amoco, and used as the matrix polymer for the membranes. Dimethylformamide, solvent for the casting solution preparation, was purchased from Duksan Pure Chemicals Co. LTD. Bovine serum albumin was bought from Merck. Whole blood was obtained from a pool of healthy volunteers at Mackay hospital (Taipei). DI-water was prepared in our laboratory with an Elga Purelab^®^ system.

### 2.2. Synthesis and Characterization of Random Copolymers

The synthesis and characterization of copolymers has been presented earlier [34] and is briefly recalled here. Styrene and 4-vinylpyridine monomers were first mixed and dissolved in DMF solvent. The molar ratio of hydrophobic styrene to hydrophilic 4-vinylpyridine was fixed to 70/30. In addition, the solid content was 30 wt%. The initiator used for the reaction, AIBN, was added to the homogeneous solution, controlling the monomer/initiator ratio to 1500. Synthesis was conducted at 70 °C for 48 h. Afterwards, the reaction was stopped by immersion of the reaction flask in ice for 30 min. The newly formed copolymer, poly(styrene-*r*-4-vinylpyridine) or P(4-*r*-4VP), was purified with water and methanol baths, and freeze-dried for 2 days.

The second part of the reaction consisted in synthesizing the zwitterionic copolymer, zP(4-*r*-4VP), from the reaction of P(4-*r*-4VP) with IPA. This reaction was performed in DMAC solvent. First, the copolymer obtained during the first part of the synthesis was dissolved in DMAC at 60 °C for 6 h. Subsequently, IPA was added. The IPA/P(4-*r*-4VP) molar ratio was 1.2, and the total solid content was controlled to 30 wt%. After mixing the compounds for 24 h, temperature was increased to 60 °C to perform the reaction. After 24 h, acetone was added to the reaction flask and stirring of the mixture was conducted for 10 min. Subsequently, zP(4-*r*-4VP) was precipitated by dropwise addition of toluene. Finally, the copolymer was washed multiple times with acetone in an ultrasonic bath until the washing solution remained clear, and it was dried under vacuum for 24 h, yielding a white material.

**Figure 2:**
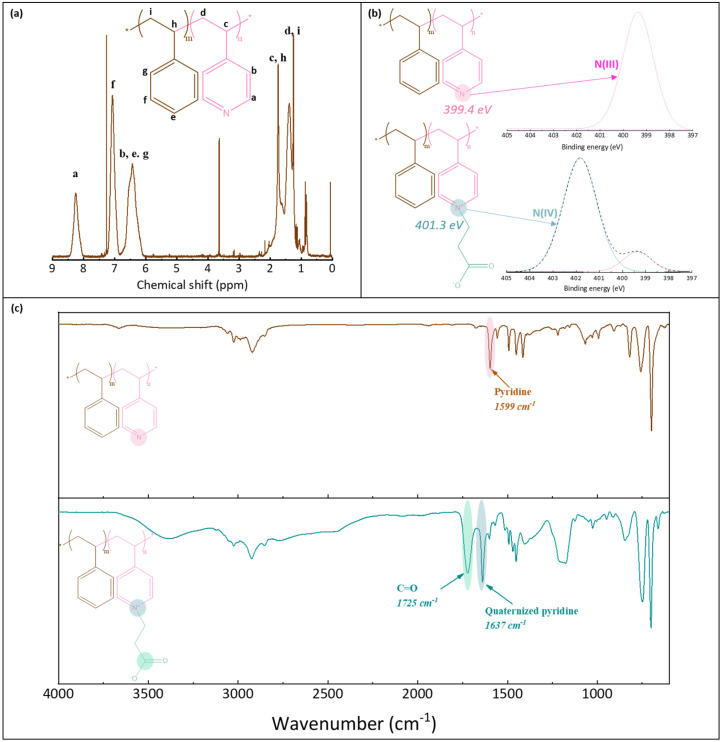
Characterization of poly(styrene-*r*-4-vinylpyridine) and zwitterionic poly(styrene-*r*-4-vinylpyridine) copolymers. (**a**) ^1^H NMR spectrum of P(S-*r*-4VP), (**b**) N1s core-level spectra of P(S-*r*-4VP) and zP(S-*r*-4VP), (**c**) FT-IR spectra of P(S-*r*-4VP) and zP(S-*r*-4VP).

^1^H NMR was used to verify the structure of P(4-r-4VP) copolymer and determine its exact styrene/4VP composition. XPS and FT-IR analyses were carried out to verify that the zwitterionization reaction was successful. ^1^H NMR tests were conducted on a Bruker 600 MHz instrument, using CDCl_3_ solvent, and a concentration of 25 mg/mL. XPS analyses were run with a PHI Quantera instrument, equipped with an AI anode producing X-rays of photon energy 1486.6 eV. Spectra were fitted using Thermo Avantage software. FT-IR analyses of the copolymer were conducted with a Perkin Elmer Spectrum One spectrometer, in ATR mode. Spectral resolution was set to 4 cm^−1^. As the copolymer used in this study was introduced earlier, only some essential characterization results associated with the synthesized batch are briefly recalled in this section for convenience of the reader (Figure 2). Peaks on the ^1^H NMR spectrum (Figure 2a) were assigned and those labelled H_a_ (on pyridine groups) and H_f_ (on styrene units) were used to determine the actual composition of the molar copolymer. It was found to be composed of 64% styrene (and so of 36% pyridine units). The molecular weight of the copolymer before zwitterionization was 108 kDa (with an index of polydispersity of 1.9). It was measured with a Viscotec instrument using both LT4000L and LT3000L columns and THF as the mobile phase (1 mL/min). XPS analysis also confirmed the successful zwitterionization reaction. While one peak only was logically found on the N1s core-level spectrum before reaction (at BE 399.4 eV), two peaks were identified on the spectrum after reaction (Figure 2b). The first small peak was also identified at about 399 eV, and corresponded to the pyridine groups, while the second larger peak was found at about 401.3 eV, and assigned to the pyridinium groups [35]. From these results, the degree of conversion of the pyridine group into pyridinium groups could be evaluated to be about 78%. Finally, the FT-IR results confirmed the successful zwitterionization reaction, as the characteristic peak of the C=O carbonyl functional group could be identified at 1725 cm^−1^, as well as that of the quaternized pyridine units at 1637 cm^−1^ (Figure 2c).

### 2.3. Casting Solution and Membrane Preparation

Casting solutions were prepared by first dissolving the zwitterionic copolymer in DMF at 60 °C. Subsequently, polysulfone was added to the solution and stirring continued at 60 °C. Complete homogenization of the system was achieved after 2 days. The polysulfone content was fixed to 20 wt%, while the zwitterionic copolymer content varied in the range 0–4 wt%. Once the solution was homogeneous, stirring was stopped for several hours before casting, to permit the removal of bubbles of gas entrapped in the viscous solution.

The viscosity of the casting solution was determined with a Viscolite 700 portable viscometer. Measurements were almost instantaneous after immersion of the instrument probe in the casting solution.

Membranes were formed by wet-immersion using a dual-bath procedure. Solutions were cast on a glass substrate, with an initial thickness of 300 µm, and immersed for 10 min in methanol. Afterwards, they were transferred to a DI water bath to complete phase inversion. After 24 h, membranes were washed with DI water to remove solvent traces, dried in air for 24 h, and stored at 4 °C until use.

### 2.4. Light Transmittance Tests

The change in light transmittance during phase inversion was monitored to highlight some potential effect of the zwitterionic copolymer on the kinetic of phase separation. Casting solutions of composition (PSf/zwitterionic copolymer/DMF: 20/4/76 wt% and 20/0/80 wt%) were prepared as detailed above. These solutions were cast with a casting knife on a glass substrate permitting the initial thickness to be controlled to 300 µm. Metallic plates were positioned on each edge of the cast solution before immersion, to avoid detachment from the substrate during film formation which could cause disturbances in light transmittance measurement. Phase inversion was conducted in a container designed in such a way that its bottom comprises a transparent section. The glass substrate on which was cast the polymeric system was immersed in the bath and carefully positioned on the transparent section. A light collector (DLM 536, Tecpel Co., Taipei, Taiwan) was positioned below the forming film, and light transmittance continuously monitored throughout phase inversion. As phase separation occurred, light transmittance gradually decreased from 100% (corresponding to the initial transparent solution) to a plateau located at about 30% (corresponding to the final film).

### 2.5. Physical Characterization of Membranes

Membranes were observed with a Hitachi scanning electron microscope (SEM) S-3000. Prior to observation, membrane samples were positioned on a SEM holder, and sputter-coated with gold. For the observations of cross-sections, the samples were fractured using liquid nitrogen. The accelerating voltage was set to 15 eV.

Porosity was determined by gravimetric measurement using butanol, a solvent that neither swells the membrane nor solubilizes the polymers. First, dry samples (of diameter 1 cm) were weighed ($W_{D}$). Subsequently, samples were immersed in the alcohol bath for 24 h. Wet samples were then weighed after gently wiping off butanol droplets from the surface of the membranes with some tissue cloth ($W_{W}$). Knowing the density of butanol ($\rho_{b}$), and of the polymer or of the mixture of polymers ($\rho_{p}$), the porosity (*ε*, %) could then be determined from the following equation:

(1) $$\varepsilon \left(\%\right)=\frac{\frac{W_{w}-W_{d}}{\rho_{b}}}{\frac{W_{w}-W_{d}}{\rho_{b}}+\frac{W_{d}}{\rho_{p}}}\times 100$$

Note that for membranes containing both polysulfone and zP(S-*r*-4VP), the density of the copolymer needed to be determined to assess the density of the polymer mixture. It was evaluated from the knowledge of the composition of the copolymer and from the molecular weight and density of each type of unit forming the copolymer. Tests were repeated 5 times.

When possible, the surface pore size was evaluated from SEM images using ImageJ^®^ software. Otherwise, the membrane pore size was calculated using the Guerout–Elford–Ferry equation as follows:

(2) $$r_{m}=\sqrt{\frac{\left(2.9-1.75\varepsilon \right)\times 8\eta lQ}{\varepsilon \times A\times \Delta P}}$$

where *ε* is the porosity found from Equation (1), *η* is the viscosity of water at the temperature of the test (taken as 0.89 × 10^−3^ Pa·s), *l* (m) is the thickness of the membrane evaluated from the SEM image of the cross-section, *Q* (m^3^/s) is the volumetric flow rate (amount of permeate collected per unit of time), *A* is the membrane surface area (m^2^), and ∆*P* (Pa) the transmembrane pressure.

### 2.6. Chemical Characterization of Membranes

The surface chemistry of membranes was characterized by XPS, ATR-FTIR, and mapping FT-IR. The XPS instrument was the same as that used to characterize the copolymer (Section 2.2). However, both the C1s and N1s core-level spectra were analyzed for the membrane systems. The FT-IR analysis was conducted with a Jasco system combining a spectroscope for ATR analyses and a microscope for mapping analyses (FT-IR 6700 and IRT-5200). Samples were first dried. ATR-FTIR analyses were run at a resolution of 4 cm^−1^. Maps were acquired over a surface of 4 mm^2^ at a wavelength centered at 1727 cm^−1^ (corresponding to the C=O function of the carboxylate group of the zwitterionic copolymer) and setting the aperture to 100 µm. At each point of the map, a spectrum was obtained by averaging 32 scans (with a resolution of 4 cm^−1^). The background for the analyses was a gold surface. Each map is color-coded. If there is no functional group, the map will be dark blue, while if there is a high density of functional groups, it will be red.

### 2.7. Characterization of Membranes’ Hydrophilic Properties

The membranes’ hydrophilic properties were determined by measuring their water contact angle (WCA) and their hydration capacity in water. For WCA tests, a DataPhysics OCA15EC instrument was used. A water droplet of a volume of about 4 µL was automatically dropped on dried samples positioned on a glass slide place on the stage of the angle meter. Subsequently, snapshots were taken and the WCA automatically measured. In this work, WCA is reported at 3 s. Ten independent tests were performed, and data reported as mean ± SD. Hydration capacity was measured by immersing dry samples (of diameter 13 mm) in DI water for 24 h. The hydration capacity corresponds to the difference per unit surface area between the wet weight of the samples (after immersion) and the dry weight (before immersion). It will be expressed in mg/cm^2^ and results of 5 independent tests are reported as mean ± SD.

### 2.8. Biofouling Tests

A number of fouling tests were performed to assess the efficiency of zP(S-*r*-4VP) to provide polysulfone membranes with biofouling resistance. Adsorption tests were carried out using *Escherichia coli* (*E. coli*) modified with a green fluorescent protein, whole blood, or bovine serum albumin (BSA). Filtration tests were carried out using BSA.

*E. coli* attachment tests were performed by incubating 1 mL of bacterial solution (whose preparation was detailed by Hsiao et al. [36]) with samples of diameter 13 mm, in a 24-well microplate. Prior to the incubation, samples were incubated with PBS overnight. Incubation of the bacterial solution with the polysulfone-based membranes was conducted for 24 h in a chamber in which the temperature was set to 37 °C. The bacterial solution was changed 3 times over the incubation period, at t = 6 h, 12 h, and 18 h, to guarantee that live microorganisms would constantly be in contact with the membrane samples. Subsequently, membranes were rinsed with PBS, and observed with a Nikon A1R confocal microscope. Three independent samples were used for each condition of membrane preparation, and 3 images analyzed for each sample. Hence the quantitative data obtained using ImageJ^®^ software correspond to an average of 9 analyses, and are reported as mean ± SD.

Whole blood attachment tests were conducted with blood obtained from healthy individuals. Samples of 13 mm were positioned in well plates, and washed with PBS at 37 °C overnight. Subsequently, PBS was replaced with 0.8 mL of whole blood. After 4 h at 37 °C in the incubation chamber, membranes were washed with PBS, and fixed with a solution of glutaraldehyde at 4 °C for 4 h, before observation with a Nikon A1R confocal microscope. As in the bacterial attachment tests, the analysis of images was carried out with ImageJ^®^ in a similar way (number of samples, images, etc.) to obtain an average (reported as mean ± SD) surface coverage of membranes by whole blood cells.

BSA adsorption tests were carried out as follows. First, dry membranes were incubated with a solution of BSA (1 g/L) for 2 h. Subsequently, membranes were scanned by mapping FT-IR using the same instrument mentioned in Section 2.6. Maps were obtained at 1650 cm^−1^, and compared to maps of membranes before incubation with the protein solution.

Filtration tests with BSA were performed in a dead-end filtration cell using circular membrane samples having a diameter of 26 mm. Samples were integrated with a non-woven support into a stirring cell. DI water was first filtered at 3.5 bar and 1100 rpm until reaching steady-state flow. Subsequently, the pressure was decreased to 3 bar, the cell loaded with 50 mL of DI water, and water flux recorded for about 30 min. Secondly, the cell was filled with 50 mL of a solution of BSA (in PBS) of concentration 1 g/L, and the flux continuously recorded until the emptying of the cell. Subsequently, the membrane was washed by immersion in DI water followed by a backflushing step using DI water and lasting 10 min. Subsequently, all operations (filtration with 50 mL of DI water, 50 mL of BSA, washing) were carried out a second time, followed by a last filtration step with pure DI water. From the knowledge of the initial water flux ($J_{w,i}$), the final protein solution flux ($J_{BSA,f}$) as recorded during the second cycle, and the final water flux as recorded at the end of the test ($J_{w,f}$), it was possible to determine essential ratios associated with fouling. These were the flux recovery ratio (FRR), the reversible flux decline ratio ($DR_{r}$), the irreversible flux decline ratio ($DR_{ir}$), and the total flux decline ratio ($DR_{t}$). Their definitions are as follows:

(3) $$FRR=\frac{J_{w,f}}{J_{w,i}}\times 100$$

(4) $$DR_{r}=\frac{J_{w,f}-J_{BSA,f}}{J_{w,i}}\times 100$$

(5) $$DR_{ir}=\frac{J_{w,i}-J_{w,f}}{J_{w,i}}\times 100$$

(6) $$DR_{t}=DR_{r}+DR_{ir}$$

## 3. Results and Discussion

### 3.1. Physical Properties of Membranes

**Figure 3:**
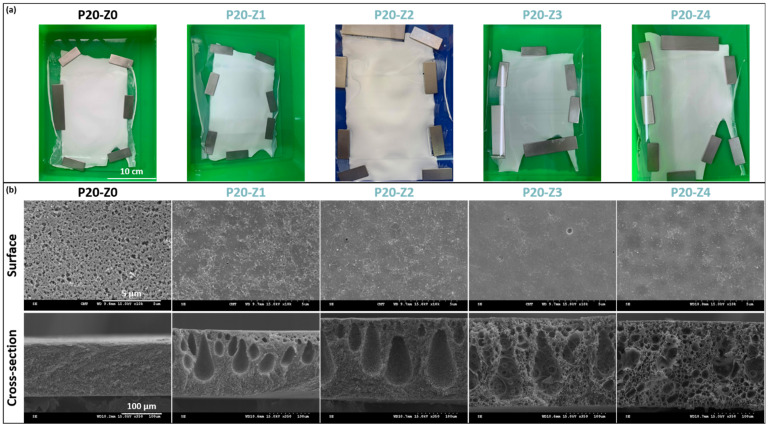
Physical characterization of membranes’ structure. (**a**) Photographs of virgin and zwitterionic membranes; (**b**) SEM analysis of surface and cross-section of virgin and zwitterionic membranes.

Membranes were hand-cast on glass plates, and the final dimensions of each membrane were at least 15 cm × 20 cm (Figure 3a). Membranes appeared whitish with a matte appearance (as opposed to shiny). Several membrane sheets were prepared for each formulation condition, from which samples were taken for further characterization.

From the matte appearance of membrane surfaces after preparation, one could conclude that porous membranes were obtained which would then be confirmed by SEM. Small pores could be seen on the membranes’ surface, although the pore size and surface porosity decreased with the copolymer content.

**Figure 4:**
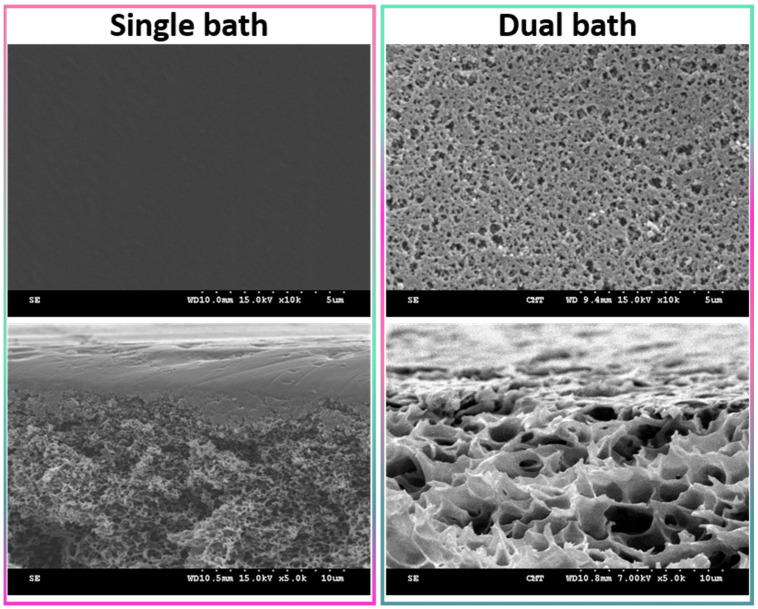
Surface of a polysulfone membrane prepared using DMF as solvent and either water as the sole non-solvent (single bath procedure) or both methanol and water as non-solvents (dual bath procedure). Top images present the surface, while the bottom images show the cross-section in the upper region.

**Figure 5:**
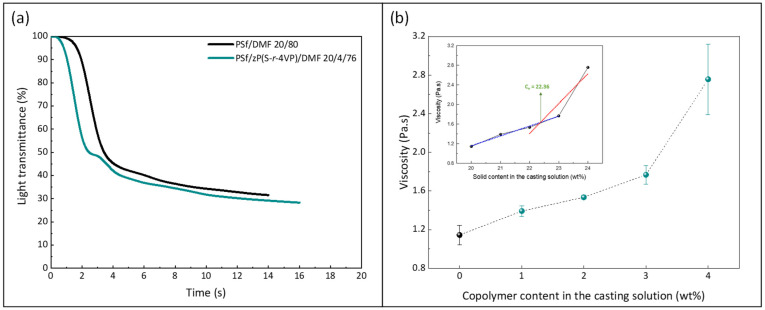
Effect of the copolymer on (**a**) kinetic of phase separation and (**b**) viscosity of the casting solution (0 wt% corresponds to the casting solution used to form the virgin membrane P20-Z0). Measurements performed at 25 °C. (Inset: determined chain entanglement concentration of the PSf/copolymer/DMF system).

Wet-immersion with DI water as the sole non-solvent, using DMF solvent for the polymer and a similar polysulfone concentration (20 wt%) led to the formation of a dense, thick skin layer (Figure 4) with a porous sublayer. The presence of this skin layer can be attributed to the gelation that has occurred as a result of the increased polymer concentration at the top layer during wet-immersion [37]. The high affinity of DMF towards water caused it to be abruptly exhausted as soon as the polymeric solution came in contact with the non-solvent (water) [38]. On the other hand, the presence of a more porous surface (P20-Z0) is likely due to the lower non-solvent power of methanol. The coarsening of the domains at the top surface observed with water as the sole non-solvent, and commonly leading to the formation of a dense skin, was partially prevented with methanol. Immersion in this weaker non-solvent enabled the retainment of the porous interface. The apparent decrease in surface pore size (as seen on the SEM image) with the addition of a copolymer can be explained by the increase in total polymer content, and so by the decrease in the free volume in the polymeric membrane. Meanwhile, cross-sectional images evidenced the presence of large pores, some similar to macrovoids in the bulk of P20-Z1, P20-Z2, and P20-Z3 membranes. They were absent from the cross-section of P20-Z0, and although large pores could still be seen in the cross-section of P20-Z4, these were not typical macrovoids. The appearance of macrovoids followed by their disappearance as the copolymer content increases can be rationalized as follows. First, the hydrophilic units of the zwitterionic copolymer tend to accelerate the penetration of non-solvent in the polymeric system during phase-inversion, as seen from the results of light transmittance tests (Figure 5a). However, adding copolymer into the casting solutions (which all contained a fixed polysulfone content) also increases the viscosity of the casting solution (Figure 5b). The latter slows down mass transfers. The presence of macrovoids is often associated with fast water penetration during film formation although some inconsistencies were pointed out in the paper of Hung et al. who stressed the need for more investigations to unify the theories on the formation of these structures [39]. Fast exchanges promote macrovoid formation. However, the use of a weak non-solvent (methanol) slowed exchanges and the formation of fingers. While phase separation and domain growth were still initiated in the first bath, these domains offered a resistance to water diffusion during immersion in the second bath. At low copolymer content (1–3 wt%), the zwitterionic units, hydrophilic, accelerated non-solvent transfers during the second immersion, and macrovoids were then observed. However, fast mass transfers were offset by the rise in viscosity in the case of P20-Z4, which would explain the disappearance of the macrovoids in this membrane. Moreover, it is noticeable in Figure 5b that the viscosity drastically increased above a certain polymer concentration which could be attributed to the entanglement of polymer chains. This phenomenon is said to occur when the amount of polymer reaches a specific threshold [39]. To determine this, the changes in the slopes of the different polymer concentrations are taken into consideration, and the intersection of these slopes can be interpreted as the threshold concentration, denoted as entanglement concentration (*C_e_*) (Figure 5b inset). From this, we determined that *C_e_* = 22.36 wt%. Hung et al. suggested that when the polymer concentration in the casting solution is above the *C_e_* and polymer chains are entangled, the starting positions (initiations) of the macrovoid growth are found below the membrane surface. Hence, in the SEM cross-section image of P20-Z3, the macrovoid growth started at the deeper part of the membrane, while in P20-Z4, the macrovoids completely disappeared, as these concentrations are above the *C_e_*. Aside from viscosity, another rheological factor that needs to be considered is the viscoelasticity of the casting solution which will be taken into account in future work in this study.

The pore size and porosity are presented in Table 1. The measured porosity, ranging between 73% and 81%, is consistent with the presence of macrovoids and large pores decorating the bulk of the membranes. The addition of amphiphilic zP(S-*r*-4VP) tends to slightly increase the matrix porosity. This effect of amphiphilic copolymers has been reported earlier [40,41]. The surface pore size could be measured from SEM images for the P20-Z0 membrane and was found to be quite large. Nevertheless, for the modified membranes, large surface pores were difficult to detect in large quantity. Thus, only the membrane pores were evaluated for these membranes using Equation (2). It is interesting to note that for P20-Z0, the membrane pores are much smaller than the surface pores, making the membrane fall in the lower region of the UF range. Modified membranes also present nanometer-scale pores, slightly larger than those of the modified membranes. Hence, although larger surface pores are hardly detected on the zwitterionic membranes, unlike the virgin membrane, they have larger bulk pores which benefit the membrane permeability.

### 3.2. Chemical Properties of Membranes

**Figure 6:**
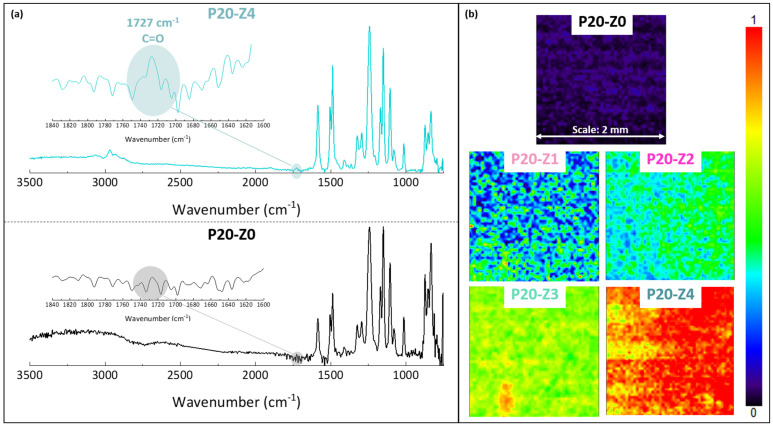
Chemical characterization of membranes by FT-IR (**a**) ATR-FTIR characteristic spectra of virgin (P20-Z0) and zwitterionic (P20-Z4) membranes. (**b**) Mapping FT-IR analysis of the different membranes, performed at 1727 cm^−1^ (C=O).

**Figure 7:**
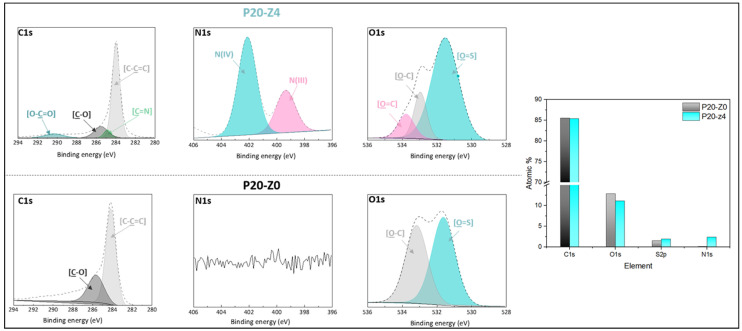
C1s, N1s, and O1s core-level spectra of P20-Z0 and P20-Z4 membranes and their corresponding elemental compositions.

The chemical composition of the membranes’ surfaces was characterized by FT-IR and XPS spectroscopy and results related to P20-Z0 (virgin) and P20-Z4 (modified) membranes, as shown in Figure 6. Although weak, the characteristic signal of C=O belonging to the carboxylate functional group of the copolymer can be detected at about 1727 cm^−1^ (Figure 6a). Thus, FT-IR can detect the presence of the copolymer on the surface of the modified membranes. Nevertheless, the XPS analysis was more helpful to evidence directly the pyridinium groups. The N1s core-level spectrum of P20-Z4 shown in Figure 7 reveals two peaks, one centered at BE 402.1 eV, corresponding to the pyridinium salt, and another one at BE 399.3 eV associated with the pyridine groups (which did not react with iodopropionic acid during the zwitterionization reaction) [34]. Logically, there is no signal seen on the N1s core level spectrum of the virgin membrane. Besides this, the analysis of the C1s core-level spectra reveals significant differences between the surface of the two membranes. Peaks of polysulfone membrane could be assigned with the help of the literature [42,43]. Subsequently, deconvolution analysis of the spectrum of P20-Z4 exposes two supplementary peaks, compared to the spectrum of P20-Z0, centered at BE 284.9 eV and 290.3 eV, corresponding to [C=N] and [O-C=O] species brought by zP(4-r-4VP), respectively. Finally, a peak centered at BE 533.8 eV and corresponding to [O=C] species can be seen on the O1s core-level spectrum of P20-Z4, associated with the carboxylate group of the zwitterionic copolymer. In addition, the elemental compositions of P20-Z0 and P20-Z4 are also illustrated. According to the XPS analysis, the surface of P20-Z4 has 2.37% nitrogen content (corresponding to the quaternized pyridine). Theoretically, we should obtain about 4%; however, it is worth noting that XPS only has a detection depth of 5 nm. Since blending entails bulk modification, some of the copolymers could be trapped inside the bulk of the PSf membranes.

A FT-IR mapping analysis of the membranes’ surfaces was also performed, to assess the level of homogeneity of the surface modification. Maps of the surfaces presented in Figure 6b are color-coded. A dark blue color signifies that the functional group of interest cannot be found while red color on the other end of the color scale means that the copolymer is present at a high density. Surfaces were mapped at 1727 cm^−1^, corresponding to the C=O functional group of the copolymer. Thus, the map corresponding to P20-Z0 (virgin membrane) is logically dark blue as this membrane does not contain any copolymer. As the copolymer content in the casting solution increased, the maps of the resulting membrane became light blue and green (P20-Z1), green with blue spots (P20-Z2), green with yellow areas (P20-Z3) and red with orange/yellow spots (P20-Z4). This change in color is logical as it supports the increasing surface density of the copolymer with its initial concentration in the casting solution. Nevertheless, it is important to note that these maps (except maybe that of P20-Z1) are constituted by one major color, or by two adjacent colors on the scale, which implies that the surfaces have reached some level of homogeneity at a large scale (scale bar is 2 mm), and so that the membrane preparation process is fairly well controlled.

All in all, these chemical analyses confirm the presence of the copolymer on the surface of the modified membranes. Therefore, changes should be detected concerning the wetting behavior and more importantly, the resistance to biofouling.

### 3.3. Hydrophilic Properties of Membranes

Hydrophilicity is regarded as one important key to the nonfouling behavior of a surface [13,44]. While superhydrophobicity can keep a membrane clean (i.e., free of dust, bacteria, proteins, etc.) when stored in air, it is not sustainable when the membrane is applied to a separation process involving some aqueous medium (as in many applications of porous membranes) because membrane wetting must occur. Thus, superhydrophobic porous membranes are ideal for a handful of applications where selective wettability (e.g., oil/water separation from oil-rich emulsion separation) is needed, but cannot really be employed for the filtration of wastewater or biological fluids. For these applications, wetting by water must occur. To protect the membrane from fouling in these environments, the membrane material should exhibit an improved affinity for water. This is because water molecules have to be expelled from the membrane material and from the biofoulant surface (e.g., protein) to reduce the free energy and make biofouling possible in aqueous media. While no (or little) control can be achieved over the hydration of the biofoulant, a proper surface or bulk modification can help strengthen the hydration layer of a membrane, and so reduce its propensity to foul.

**Figure 8:**
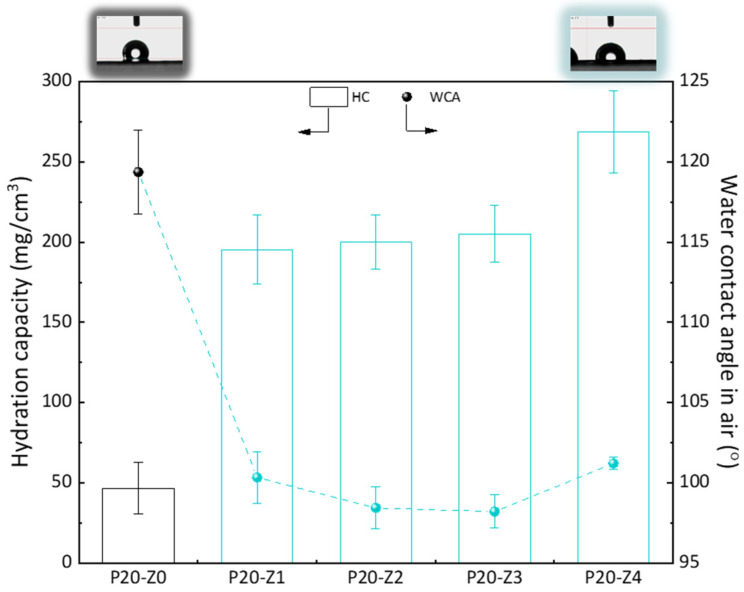
Hydrophilic properties of membranes.

Here, we chose to modify the polysulfone membrane with a zwitterionic copolymer via *in-situ* modification. Although surface segregation occurs during the phase inversion of such membranes prepared by blending a hydrophobic polymer with a more amphiphilic copolymer [45], not all molecules of the copolymer can be found at the surface. Thus, compared to a surface modification process (grafting, coating), the surface properties, including the water contact angle (WCA) in air, cannot be importantly modified. It is seen here in Figure 8 that the WCA in air of the modified membranes was about 100°, while it was 120° for the virgin membrane. While this does show some improvement of the hydrophilicity of the surface, this is certainly not low enough to prevent fouling of the membrane surface in water. However, when the membrane is used in separation, it is usually immersed for some time in the aqueous environment. Wetting may take time but does occur when the amphiphilic material has been blended with the hydrophobic polymer. The WCA in air was measured a few seconds after dropping water on the surface of the membrane, and so it does not represent the actual affinity of the modified membranes with water when contacted with the liquid environment for a time long enough to establish hydrophilic interactions. Hydration capacity (HC) measurements, however, involve a 24-h immersion of the membrane material with DI water. The results of Figure 8 show an important increase in HC when comparing the virgin membrane to the modified ones. While it was measured to be about 50 mg/cm^3^ for the virgin membrane, it almost reached 275 mg/cm^3^ for P20-Z4 membrane. This result proves that water molecules can be more easily trapped in the bulk of the modified membrane, physically (in the pore) or chemically (interactions with the zwitterionic heads), because of the modification of the casting solution with the amphiphilic copolymer. Hence, the modified membranes should be less prone to fouling than the virgin PSf membrane.

### 3.4. Effect of the Zwitterionic Copolymer on Resistance to Biofouling by Escherichia coli Bacteria

Bacteria are commonly found in wastewater [46] and the presence of these pathogens may pose a threat to the environment and to people. Pathogenic *Escherichia coli* have been identified in sewage treatment plants [47]. Removal of these pathogens can be achieved by porous membranes, but biofouling of the membrane material by these microorganisms can quickly occur [48], resulting in a severe decrease in permeability. Ideally, a membrane surface should resist bacterial attachment to mitigate the effect of biofilm formation.

**Figure 9:**
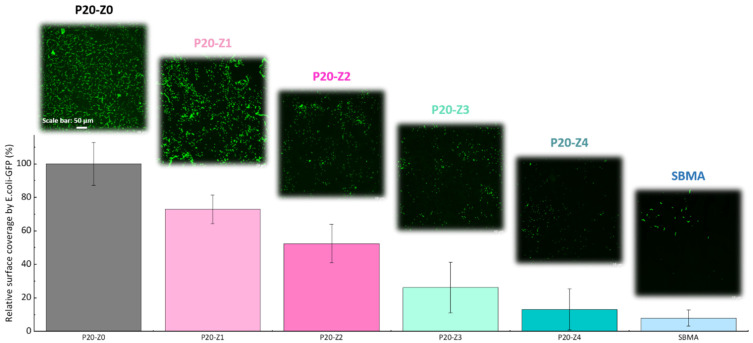
Effect of zwitterionic copolymer on the resistance of membranes to the attachment of *Escherichia coli*.

In this study, the effect of zP(4-*r*-4VP) copolymer on the resistance of polysulfone membranes to bacterial attachment was tested after incubation of the membrane for 24 h with a solution of *E. coli*. As seen in Figure 9, the virgin membrane (P20-Z0) is covered by bacterial species. Biofouling of polysulfone membranes by *E. coli* is a well-known issue for these membranes that has been reported in the literature many times [26,49], and can be rationalized by the formation of hydrophobic–hydrophobic interactions between the substrate and the cells. The addition of zwitterionic copolymer to the initial casting solution results in an important decrease in bacterial attachment. Hence, our results show that bacterial attachment on the P20Z4 membrane fell to less than 15% (compared to the virgin membrane), and the antibiofouling property of P20-Z4 was almost comparable to that of the sulfobetaine methacrylate (SBMA) hydrogel control, a common antifouling material [50,51,52]. The zwitterionic copolymer promotes water trapping and the formation of a hydration layer which is thought to protect the membrane from biofouling. The results of Figure 8 reveal a drastic increase in hydration capacity. Water trapped after prolonged immersion in some aqueous medium (as during bacterial attachment tests) protects the polymer matrix from biofouling by preventing hydrophobic interactions. Chen and coworkers [53] stressed the importance of hydration to successfully design surfaces resistant to proteins or bacteria. Wu and coworkers demonstrated that one unit of PSBMA zwitterionic material could tightly bound with eight molecules of water, which explains its excellent resistance to nonspecific protein adsorption and cell adhesion [54]. To the best of our knowledge, a similar investigation has not been carried out with the zwitterionic material at play in this study. However, it seems reasonable to state that the successful resistance of zP(S-*r*-4VP)-modified membranes to bacterial attachment also lies in the material’s ability to promote hydration.

### 3.5. Effect of the Zwitterionic Copolymer on Resistance to Biofouling by Whole Blood

Polysulfone is commonly used as a base material for the design of hemodialysis membranes [5,55,56]. The study of Wenten and coworkers emphasized the need for a modification process in view of reducing blood protein adsorption that then leads to platelet adhesion and activation [56]. One highly efficient system is that presented by Ishihara et al. [57]: 2-methacryloyloxyethyl phosphorylcholine (MPC) polymer can drastically reduce the interactions of fibrinogen and platelets with a polysulfone membrane system, and hence improve its blood compatibility. The downside is related to the cost associated with the synthesis of MPC. Nevertheless, and based on these previous studies, the zwitterionization of polysulfone membranes by the *in-situ* modification method is a viable technique to improve the hemocompatibility of the membranes.

**Figure 10:**
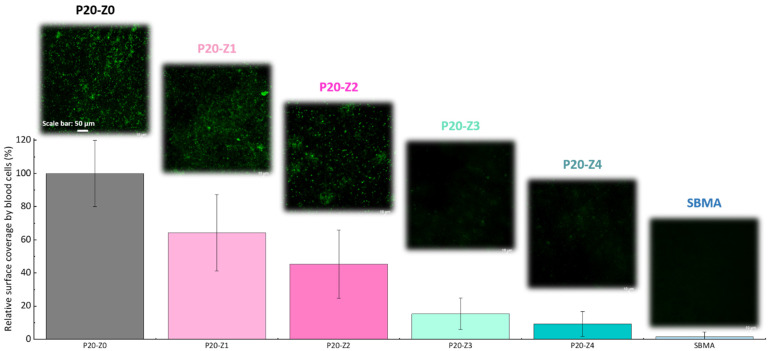
Effect of zwitterionic copolymer on the resistance of membranes to the attachment of cells from whole blood.

Here, we challenged the membranes against whole blood. As in the bacterial attachment test, membranes were incubated with whole blood and their surface observed by confocal microscope after fixing/staining the cells. The results provided in Figure 10 (1) demonstrate the efficiency of the copolymer in improving the blood compatibility of polysulfone membranes, (2) confirm that the blending method can be an efficient means to improve the blood compatibility of hydrophobic membranes and (3) show that despite not being entirely zwitterionic, unlike the SBMA control, P20-Z4 almost competes with purely zwitterionic hydrogel materials. These results could not have been obtained without a homogeneous and dense distribution of the copolymer on the surface of the membrane (Figure 8). The use of methanol as a first non-solvent bath likely facilitated diffusion of the copolymer chains leading to surface segregation (compared to the use of pure water which would lead to faster phase inversion and so faster solidification of the polymeric domains). Ultimately, it contributed to the excellent resistance to blood.

### 3.6. Effect of the Zwitterionic Copolymer on Resistance to the Adsorption of Bovine Serum Albumin Protein

The attachment of cells onto a membrane is often triggered by the earlier adsorption of proteins, smaller biofoulants, but the absence of cells does not indicate that the material surface is nonfouling. Biofouling by proteins occurs at a smaller scale than biofouling by cells, so it is harder to prevent. Pape et al. recently showed that films with non-cell-adhesive properties still displayed significant levels of protein adsorption [58]. Numerous techniques can be used to quantitatively or qualitatively assess protein adsorption on a material surface. Benavente et al. reported that mapping FT-IR was a powerful technique to evaluate the resistance to proteins of antifouling membranes [59], at relatively large scale (mm). Here, we used a similar method to study the adsorption of BSA on the surface of P20-Z0 and P20-Z4 membranes. Analysis was carried out at 1650 cm^−1^, corresponding to the amide I functional group of the protein [60].

**Figure 11:**
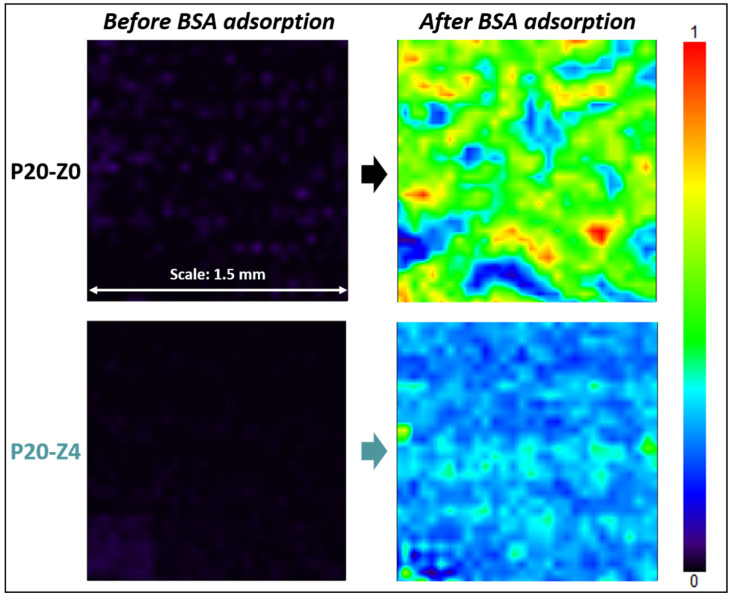
Effect of the additive on BSA adsorption. FT-IR maps at 1650 cm^−1^ of P20-Z0 and P20-Z4 before and after incubation with BSA solution.

Results shown in Figure 11 highlight that the zwitterionic copolymer significantly reduced protein adsorption. While the map of the modified membrane (P20-Z4) was dominated by a light blue color after the incubation of the samples with the protein solution, that of the virgin membrane was mostly green. The light blue color indicates that the adsorption of BSA on the membrane surface is low according to the color-code. On the other hand, as the map turns green with orange and red spots, the adsorption of the protein is much more significant. A complementary quantitative test led to an adsorption level of 0.13 mg/cm^2^ (±0.01 mg/cm^2^, *n* = 3) after immersion of the P20-Z0 membrane in a 1 g/L BSA solution for 2 h, and UV/Vis spectrophotometry measurements. This adsorption level could easily be reduced by 60% on P20-Z4 membrane, which indicates that the copolymer can also mitigate the biofouling propensity of small biomolecules. This is important as these biomolecules often trigger biofouling by larger biofoulants (cells).

### 3.7. Effect of the Zwitterionic Copolymer on Resistance to Biofouling in Dynamic Conditions

**Figure 12:**
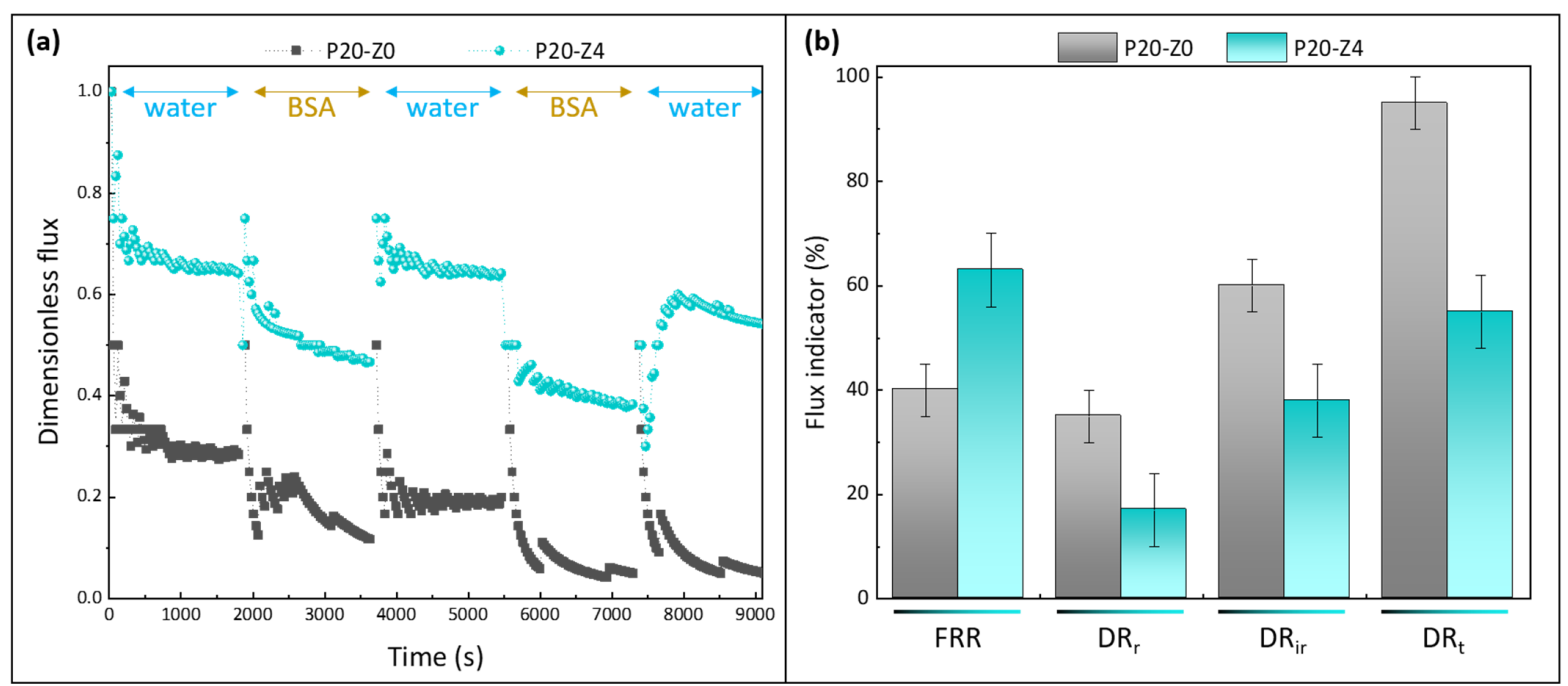
Effect of zwitterionic copolymer on the resistance of membranes to protein fouling during water/BSA cyclic filtration. (**a**): dimensionless flux; (**b**): flux indicator ratios (FRR: flux recovery ratio; DR_r_: reversible decline ratio; DR_ir_: irreversible decline ratio; DR_t_: total decline ratio). *n* = 3 independent tests were carried out.

Membrane resistance to biofouling was also evaluated in dynamic conditions (as opposed to attachment tests that were static) by carrying out filtration tests with a protein solution. Bovine serum albumin is classically used to study membrane fouling during filtration, whether membranes fall in the microfiltration domain [61,62,63,64], or in the ultrafiltration range [65,66]. Here, filtration was carried out in dead-end mode. This configuration promotes the interactions of biofoulant materials with the membrane material by orienting the feed flux directly towards the membrane surface. Hence, short-term tests are sufficient to compare the antifouling performances of membranes. Tests were run with the pristine PSf membrane (P20-Z0) and the zwitterionic PSf membrane (P20-Z4), and results shown in Figure 12. The purpose of these tests was not to focus on rejection but to study the effect of the copolymer on the antifouling property during filtration. However, it is worth noting that the rejection after one cycle was 80.8 ± 0.6% using P20-Z0, while it increased to 95.6 ± 2.2% using P20-Z4 (measured from three independent tests).

Permeability results were normalized to be able to compare at first glance the performances of the membranes (Figure 12a). For the virgin membrane, the initial flux was about 17 LMH at 3 bar, which is close to the findings of Madaeni and Rahimpour (18 LMH at 4 bar) [67]. This low permeability can readily be explained by the fact we did not use any pore-forming agent to prepare the virgin membrane, unlike in most reports [31,32,33]. For example Xu et al. used as much ethylene glycol monomer (16 wt%) as there was polysulfone to prepare the virgin membrane [33]. Here, it was decided to not use any pore-forming agent to be able to evaluate the actual effect of the copolymer (thus, the prime objective was not to reach high flux). The permeability of the modified membrane was found to be 35 LMH at the same transmembrane pressure; that is, a twofold increase was obtained thanks to the zwitterionic copolymer. As seen earlier, the copolymer not only modified the surface and bulk structure (physical effect), but also changed the membrane surface/bulk chemistry. Enlarging the pores in the cross-section benefited water permeability. Altering the bulk chemistry by adding zwitterionic units also acted in favor of faster transport. A similar effect of amphiphilic copolymers blended with hydrophobic polysulfone has been reported [68].

The copolymer clearly improved the flux recovery ratio of the membranes, averaging at about 63% (±5%) at the end of the test using the modified membrane P20-Z4, while it was measured to be 40% using only the virgin membrane P20-Z0. Notably, the copolymer reduced irreversible fouling by BSA during the filtration procedure (from 60% for P20-Z0 to 38% for P20-Z4), which impacted the total flux decline. Thus, its effect was not only noticeable in static adsorption tests (Figure 11) but also during dynamic tests carried out in dead-end filtration, the filtration configuration in which fouling occurs the fastest. It is also important to note that the higher rejection measured with P20Z4, compared with P20Z0, is a consequence of smaller surface pore size and of the presence of antifouling units decorating the membrane surface which partially prevent protein adsorption and penetration in the bulk. Thus, despite a larger membrane pore size (10.4 nm vs. 6.3 nm), the repelling effect of the copolymer permitted an increase in the protein rejection.

### 3.8. Assessment of the Modified Membranes’ Stability—Directions to Explore to Improve the Design

**Figure 13:**
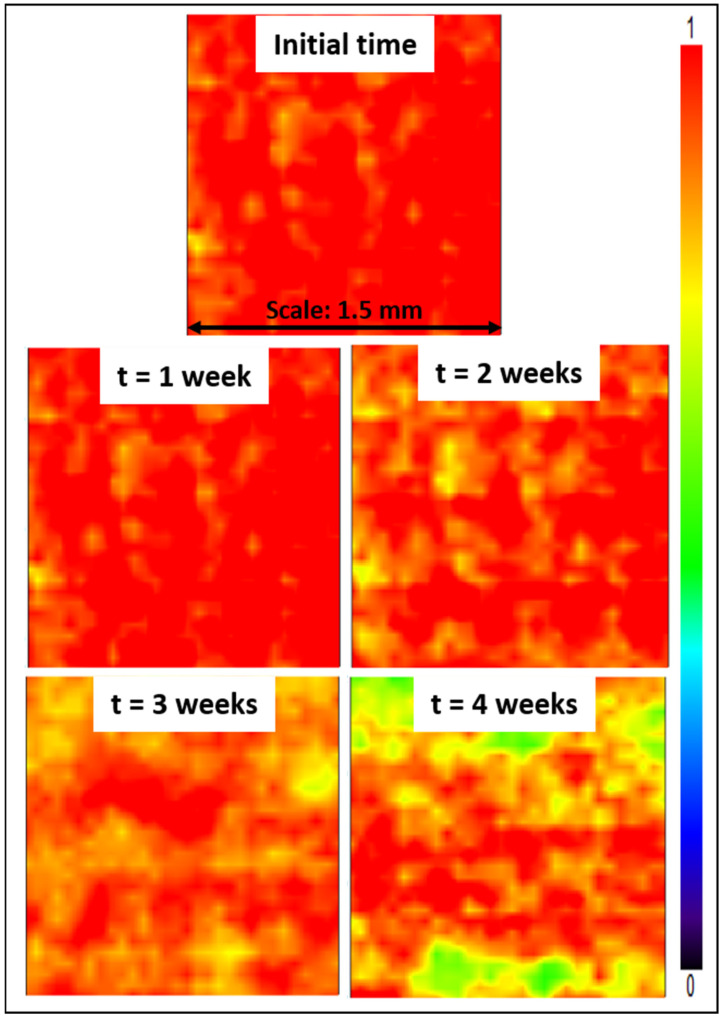
Mapping FT-IR images at 1727 cm^−1^ before and after immersing the membranes in DI water for 4 weeks.

Lastly, stability tests were conducted, consisting in immersing Z4 membranes for several weeks in a DI water bath. Subsequently, mapping FT-IR analyses were conducted. The purpose was to study the effect of the long-term immersion on the zwitterionic copolymer release from the membrane surface. The stability of membrane modification relies on hydrophobic–hydrophobic interactions in the case of *in-situ* modification (i.e., initial blending followed by phase-inversion). The hydrophobic forces are challenged by hydrophilic forces at play between the zwitterionic units of the copolymer and water from the aqueous environment in which membranes are immersed. These hydrophilic forces may result in the leaching of copolymer molecules, and so in a gradual loss of antifouling properties. Partial leaching of the zwitterionic copolymer from the polysulfone membrane could be detected from the results of Figure 13, as a change in color associated with a change in copolymer surface density. Nevertheless, it is undeniable that a large proportion of copolymer still remained trapped in the bulk of the membrane after 4 weeks, with a dominating orange-red color. Comparing these data with those presented earlier in Figure 6, the surface of the Z4 membrane immersed in DI water for several weeks still displayed a higher copolymer density than the surface of the Z3 membrane. Thus, the loss of material, though detectable, remains small and controllable.

The results gathered and evaluated in this study and from our previous studies [30] indicate that the zwitterionic poly(styrene-co-4-vinylpyrridine) copolymer exhibits good versatility in terms of being able to provide effective antifouling properties on PVDF-based membranes. However, efforts can still be made to fully materialize the efficiency of these antifouling membranes. On this note, several directions are now being investigated in our team to improve further the stability of the membranes. More details are provided as follows.

- Increasing the length of the hydrophobic segments, i.e., augmenting the relative proportion of styrene units, would be the most evident method as it would permit the strengthening of the stabilizing hydrophobic interactions (and in the meantime weaken the hydrophilic interactions).
- Reducing the zwitterionic degree of the 4VP units (from 78% as in this work to a lower value) may provide a good tradeoff between the reduction of destabilizing hydrophilic interactions and the preservation of antifouling properties.
- One could also consider changing the copolymer configuration. While we worked here with a random copolymer (it can be readily synthesized at relatively low costs), a block copolymer may lead to better stability and antifouling performances, although it would be more challenging to synthesize. In block configuration, all hydrophobic units would be entangled in the matrix, while most hydrophilic units would be found at the interface between the membrane and the surrounding environment. In other words, each unit would fulfill the function it was originally intended for. In random configuration, some isolated hydrophobic units surrounded by numerous hydrophilic units may not be entangled in the membrane and conversely, some hydrophilic units may be found trapped in the main polymer matrix.

## 4. Conclusions

Polysulfone membranes, modified with a zwitterionic copolymer obtained after a reaction of iodopropionic acid with poly(styrene-co-4-vinylpyrridine), were prepared by a dual-bath procedure. The goal was to evaluate the effect of the copolymer on membrane structure, arising properties, and its ability to mitigate fouling. After incubating the membranes with *E. coli*, biofouling was reduced by about 87%, compared to the virgin membrane. Similarly, biofouling by whole blood was dramatically mitigated (90% reduction) as the adsorption level of blood cells on the P20-Z4 membrane (containing 4 wt% additive) was almost comparable to that measured using a control zwitterionic poly(sulfobetaine methacrylate) hydrogel. Biofouling tests involving BSA protein also demonstrated the ability of the membranes to mitigate biofouling at the nano scale over large surfaces (as seen from a mapping FT-IR analysis). A flux recovery ratio of 63% was obtained after cyclic BSA/water filtration tests with the modified membranes, while it was measured at 40% with the virgin membrane.

The present membranes are stable for several weeks and provide enhanced antibiofouling properties against a variety of biofoulants. Although the design can be improved to reach a better tradeoff between permeability, stability, and biofouling-resistance, it is believed that these membranes could be suitable for applications in the biomedical field where long-term stability is rarely needed (as membranes are often discarded after single use); high permeability is not the main focus, but biofouling-resistance is essential to prevent cell lysis.

## Figures and Tables

**Table 1:** Porosity and pore size of the virgin and modified PSf membranes. ^(1)^ Evaluated when possible from SEM, ^(2)^ Evaluated from Equation (2).

Membrane ID	Porosity (%)	Surface Pore Size (μm) ^(1)^	Mean Pore Size (nm) ^(2)^
P20-Z0	73.2 ± 1.4	0.9 ± 0.3	6.3
P20-Z1	77.0 ± 1.5	/	9.1
P20-Z2	80.2 ± 0.5	/	10.4
P20-Z3	79.2 ± 0.5	/	10.3
P20-Z4	74.1 ± 1.5	/	10.4

## Data Availability

Not applicable.
